# Tumour-necrosis factor-A polymorphisms and gastric cancer risk: a meta-analysis

**DOI:** 10.1038/sj.bjc.6604277

**Published:** 2008-03-04

**Authors:** F Gorouhi, F Islami, H Bahrami, F Kamangar

**Affiliations:** 1 Department of Medicine, The Reading Hospital and Medical Center, West Reading, PA, USA; 2 Digestive Disease Research Center, Medical Sciences/University of Tehran, Tehran, Iran; 3 International Agency for Research on Cancer, Lyon, France; 4 Departments of Medicine and Epidemiology, Johns Hopkins Schools of Medicine and Public Health, The Johns Hopkin's University, Baltimore, MD, USA; 5 Division of Cancer Epidemiology and Genetics, National Cancer Institute, National Institutes of Health, Bethesda, MD, USA

**Keywords:** TNF, gastric cancer, polymorphism, inflammation, genetic

## Abstract

Inflammation is one of the early phases in the development of gastric cancer. Therefore, several studies have examined the association of polymorphisms in tumour-necrosis factor-A gene (*TNF-A*) with gastric cancer risk. This meta-analysis reviews and summarises published evidence for these associations. Searching several databases yielded 24 independent studies that reported on the associations between *TNF-A* polymorphisms and gastric cancer risk. We analysed available data for the most commonly investigated polymorphisms: *TNF-A* –308G>A (23 studies), *TNF-A* –238G>A (9 studies), and *TNF-A* –857C>T (5 studies). Summary odds ratios (ORs) and 95% confidence intervals (95% CIs) were calculated in the random-effects model using the DerSimonian–Laird method. *Q*-statistic and *I*^*2*^-statistic were calculated to examine heterogeneity, and funnel plots were plotted to examine small study effects. The overall ORs (95% CIs) for AG and AA genotypes *vs* GG genotype for *TNF-A* –308 were 1.09 (0.94–1.27) and 1.49 (1.11–1.99), respectively. For *TNF-A* –238, the corresponding ORs (95% CIs) were 1.05 (0.84–1.33) and 1.25 (0.30–5.26), respectively. The overall ORs (95% CIs) for CT and TT genotypes (*vs* CC) for *TNF-A* –857 were 1.06 (0.89–1.27) and 1.57 (0.91–2.70), respectively. The statistically significant association between *TNF-A* –308GG and gastric cancer was limited to western populations. This association showed little heterogeneity (*I*^*2*^=0) and remained consistently strong when analyses were limited to anatomic and histologic subtypes of gastric cancer, or limited to studies in which genotype frequencies were in Hardy–Weinberg equilibrium, or limited to larger studies. These same subgroup analyses did not change results associated with other polymorphisms. In conclusion, *TNF-A* –308AA genotype was associated with a statistically significant increased risk of gastric cancer, whereas other studied polymorphisms were not. The association between *TNF-A* –857TT genotype and gastric cancer was near significant, and may become significant if more studies are published.

Inflammation in the form of chronic superficial gastritis is one of the early phases in the development of gastric cancer (Correa, 1992). Therefore, a stronger inflammatory response by the host may modify gastric cancer risk. The first published epidemiologic evidence for this hypothesis came from a study published in *Nature*, which showed an increased risk of gastric cancer and its precursor lesions associated with proinflammatory polymorphisms in interleukin-1B (*IL-1B*) and *IL-1RN* genes (El Omar *et al*, 2000). Subsequent studies presented more data on the associations between several proinflammatory polymorphisms and gastric cancer. Some studies suggested that these polymorphisms increase gastric cancer risk, and, in some studies, this increase in risk was observed only in certain subgroups such as certain anatomical subsites (ie, noncardia) (El Omar *et al*, 2003) or histological subtypes (ie, intestinal type) (Machado *et al*, 2001) of gastric cancer. Other studies, however, showed no association or even inverse associations.

In addition to polymorphisms in interleukin genes, the polymorphisms in the promoter region of tumour-necrosis factor-A (*TNF-A*) gene have been extensively studied in relation to gastric cancer. Three polymorphisms in *TNF-A* genes have been studied more than the other polymorphisms. *TNF-A* –308G>A is associated with an increased production of TNF-*α* (Jang *et al*, 2001), which is a central mediator of the immune response and shares many biologic properties with IL-1. The function and significance of *TNF-A* −238G>A is less clear, but because a putative repressor site is located in a 25-base stretch that includes position −238, this polymorphism may be functional (Jang *et al*, 2001). *TNF-A* −857C>T is also associated with higher transcriptional activity of *TNF-A* (Hohjoh and Tokunaga, 2001).

Since the previous results have been inconclusive regarding the associations between *TNF-A* genotypes and gastric cancer risk, the purpose of this meta-analysis is to review studies that have examined those polymorphisms. Where possible, we examine these associations by anatomical or histological subtypes of gastric cancer, and by *Helicobacter pylori* positivity.

## METHODS

### Selection of studies

We conducted a comprehensive search by examining several databases for all papers that had been published on the association between *TNF-A* polymorphisms and gastric cancer risk. All results were updated on 15 October 2007. The following terms were used in PubMed Databases search: (‘Interleukins’ [MeSH] OR ‘Tumor Necrosis Factor-alpha’ [MeSH] OR (Tumor Necrosis) OR TNF) AND (‘Stomach Neoplasms’ [MeSH] OR (gastric cancer) OR (stomach cancer)) AND (‘Polymorphism, Genetic’ [MeSH] OR polymorphism OR polymorphisms). The following terms were used in ISI Database search: (TS=(Interleukins) OR TS=(Tumor Necrosis Factor-alpha) OR TS=(Tumor Necrosis) OR TS=(TNF)) AND (TS=(Stomach Neoplasms) OR TS=(gastric cancer) OR TS=(stomach cancer)) AND (TS=(Polymorphism, Genetic) OR TS=(polymorphism) OR TS=(polymorphisms)). Other databases and search terms were MedCarib, LILACS, IMEMR, IndMed, and PAHO databases, searched for (gastric OR stomach) AND (cancer OR carcinoma OR neoplasms); IMSEAR database, searched for combinations of gastric or stomach with cancer or carcinoma or neoplasms; and J-EAST database, searched for combinations of gastric or stomach with cancer or carcinoma or neoplasms plus polymorphism. In addition, references of cited articles were reviewed.

Two of the authors reviewed results of each of the database searches to make sure that published papers are not missed. In addition, where overall data were missing, we contacted the authors for further information.

Using these approaches, reports on *TNF-A* polymorphisms in relation to gastric cancer was found in a total of 29 articles (Jang *et al*, 2001; Wu *et al*, 2002, 2003, 2004; El Omar *et al*, 2003; Garza-Gonzalez *et al*, 2003, 2005; Machado *et al*, 2003; Fei *et al*, 2004; Glas *et al*, 2004; Lee *et al*, 2004, 2005; Ohyama *et al*, 2004; Torres *et al*, 2004; Guo *et al*, 2005; Li *et al*, 2005; Lu *et al*, 2005; Perri *et al*, 2005; Rocha *et al*, 2005; Zambon *et al*, 2005; Kamangar *et al*, 2006a; Kim *et al*, 2006; Morgan *et al*, 2006; Shirai *et al*, 2006; Deans *et al*, 2007; Garcia-Gonzalez *et al*, 2007; Hou *et al*, 2007; Seno *et al*, 2007; Sugimoto *et al*, 2007). Three studies (Garza-Gonzalez *et al*, 2003; Wu *et al*, 2003; Shirai *et al*, 2006) were excluded from the analyses because their results were reported in other studies (Ohyama *et al*, 2004; Wu *et al*, 2004; Garza-Gonzalez *et al*, 2005). One more study was excluded because the results had been reported for a combination of oesophageal and gastric cancers (Deans *et al*, 2007). Another study (Seno *et al*, 2007) was excluded because genotype frequencies were not reported. Therefore, a total of 24 studies were used for calculating summary statistics.

### Data extraction and statistical analysis

For *TNF-A* −308 (rs1800629) and *TNF-A* −238 (rs361525), numbers and percentages of GG, GA, and AA genotypes, and for *TNF-A* −857 (rs1799724), numbers and percentages of CC, CT, and TT genotypes were extracted by case status. For GG and GA *vs* GG genotypes (*TNF-A* −308 and −238) and for TT and TC *vs* CC genotypes (*TNF-A* −857), odds ratios (ORs) and 95% confidence intervals (95% CIs) were calculated. We performed similar calculations for AA *vs* a combination of GA and GG genotypes (*TNF-A* −308 and −238), and for TT *vs* a combination of TC and CC genotypes (*TNF-A* −857) to examine the applicability of recessive models. Likewise, we did similar analyses for a combination of AA and GA *vs* GG genotypes (*TNF-A* −308 and −238) and for combination of TT and TC *vs* CC genotypes (*TNF-A* −857) to examine whether dominant models apply. We used both random-effects models (DerSimonian–Laird method) and fixed-effects models (Mantel–Haenszel method) to calculate overall summary ORs and 95% CIs. Because these two methods yielded similar results, we chose only random-effects models (Moayyedi, 2004) to present forest plots, and all other analyses described from here onwards.

Some of the published studies found associations only with certain anatomical subsites (ie, noncardia) or histological subtypes (ie, intestinal type) of gastric cancer. Therefore, we calculated summary ORs and 95% CIs for noncardia cancer, where genotype data were presented by anatomical location, and for intestinal-type cancer, where data on histology were available. We examined the association between *TNF-A* −308 and gastric cancer in studies that reported this association among *H. pylori*-positive subjects. It has been suggested that proinflammatory polymorphisms in interleukins may be associated with higher risk of gastric cancer in western countries, but not in East Asian countries. Therefore, study populations were classified as western (Europe and Americas) *vs* East Asian (China, Korea, Taiwan, and Japan), and subgroup analyses were performed for each group. In all, 12 studies were from western and 12 studies were from East Asian countries.

We examined the effect of Hardy–Weinberg equilibrium (HWE) on the results of our meta-analysis by calculating summary ORs and 95% CIs for studies in which these alleles were in HWE among controls. The strategy was to exclude studies in which genotypes violated HWE at *α*=0.05.

We plotted Begg's funnel plot to examine small study effects (Sterne *et al*, 2001). We also used the method of Begg and Mazumdar (1994) to calculate *P* for rank correlation and Egger's weighted regression method (Egger *et al*, 1997) to calculate *P* for bias. For sensitivity analysis, we excluded smaller studies and recalculated the summary ORs (95% CIs) using only larger studies.

To examine result heterogeneity among studies, the *Q*-statistic for homogeneity (using Mantel–Haenszel weights) and the *I*^*2*^-statistic (Higgins *et al*, 2003) were calculated. All analyses were done using STATA software, version 9.2 (STATA Corporation, College Station, TX, USA). Throughout the paper, two-sided *P*-values <0.05 were considered as statistically significant.

## RESULTS

Twenty-four studies with a total number of 4399 cases and 6855 controls were included in this analysis (Table 1). The most commonly investigated genotypes were *TNF-A* −308, −238, and −857, which were reported in 23, 9, and 5 studies, respectively. Since other genotypes, such as *TNF-A* −1031, were investigated in a very small number of studies, only data on the above mentioned three genotypes were analysed. Most studies used healthy volunteers or blood donors as control subjects. The frequency of *TNF-A* −308A and *TNF-A* −238A alleles ranged from 0.9 to 16.2% and from 1.2 to 10.3%, respectively. The frequency of *TNF-A* −857T allele ranged from 14.3 to 19.6%. Median frequencies of *TNF-A* −308A allele were 12.5 and 6.8% in western populations and East Asian populations, respectively. Corresponding frequencies for *TNF-A* −238A allele were 4.9 and 3.8%, and for *TNF-A* −857T allele were 19.6 and 15.0%, respectively.

### *TNF-A* −308

**Figure 1:**
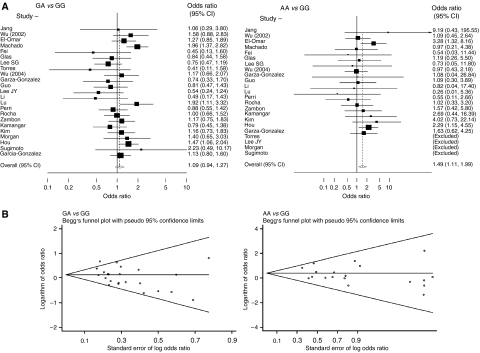
The association between *TNF-A* −308 and gastric cancer (GA and AA genotypes *vs* GG). (**A**) Forest plot – studies are sorted in order of publication year. (**B**) Begg's funnel plots for the associations.

Study-specific and summary ORs (95% CIs) are shown in Figure 1A. For all gastric cancers, the random-effect overall ORs (95% CIs) associated with heterozygous (GA *vs* GG) and homozygous (AA *vs* GG) proinflammatory genotypes were 1.09 (0.94–1.27) and 1.49 (1.11–1.99), respectively, and the corresponding fixed-effect ORs (95% CIs) were 1.14 (1.02–1.27) and 1.51 (1.14–1.99), respectively. Recessive model was the best-fitting inheritance model for *TNF-A* −308. However, because GG genotype was much more frequent than GA and AA genotypes, comparing GA+GG *vs* AA genotype did not have a material effect on the test power. Therefore, the results are reported separately for GA *vs* GG and AA *vs* GG genotypes.

Table 2 summarises overall and subgroup-specific summary ORs and 95% CIs. Only a fraction of studies presented data on subgroups. For example, only 6 out of the 23 studies reported their results by intestinal *vs* diffuse histology types. Among these, summary ORs (95% CIs) for GA *vs* GG and AA *vs* GG genotypes were 1.17 (0.85–1.59) and 2.88 (1.74–4.77) for noncardia cancers and 0.98 (0.68–1.40) and 2.17 (1.17–4.03) for intestinal-type gastric cancers, respectively. When we limited our analysis to *H. pylori*-positive cases and controls, these ORs (95% CIs) were 0.66 (0.33–1.30) and 3.23 (0.10–99.4), respectively. The summary ORs (95% CIs) for GA *vs* GG and AA *vs* GG genotypes were 1.14 (0.95–1.37) and 1.74 (1.21–2.51) for western countries, and 1.02 (0.78–1.34) and 1.14 (0.70–1.84) for eastern countries, respectively.

In three studies, the distributions of *TNF-A* −308 genotypes among controls were not in HWE (Table 1). Limiting the analysis to the studies with HWE, the summary ORs (95% CIs) for GA *vs* GG and AA *vs* GG genotypes were 1.08 (0.91–1.28) and 1.73 (1.22–2.44), respectively.

Figure 1B shows Begg's funnel plot for the association between *TNF-A* −308 and gastric cancer. This figure shows logarithm of OR (*Y* axis) *vs* its standard error (SE) (*X* axis). Smaller studies have larger SEs, therefore points showing these studies are in the right-hand side of the graph. For GA *vs* GG genotypes, there was evidence for bias using Egger's weighted regression method (*P* for bias=0.03) and using the method of Begg and Mazumdar (*P*=0.07). There was no evidence of bias for AA *vs* GG genotypes using either Egger's method (*P* for bias=0.44) or Begg's method (*P*=0.87). We excluded small studies, defined as those having an SE>0.5. After these exclusion, the summary OR (95% CI) changed to 1.13 (0.97–1.32) for GA *vs* GG, and 1.68 (1.08–2.61) for AA *vs* GG.

Table 2 shows the *Q*- and *I*^*2*^-statistics for the overall and subgroup analyses. For the overall analysis, the *Q*-statistic was significant (*P*=0.02) and *I*^*2*^ (34%) showed a moderate variation for GA *vs* GG genotypes. For AA *vs* GG genotypes, the *Q*-statistic was not significant (*P*=0.74) and *I*^*2*^ was equal to zero. Subgroup analyses showed similar patterns as overall analyses. While for most subgroup analyses, the *P*-value for GA *vs* GG genotypes was significant or remained close to significance level and *I*^*2*^ showed moderate heterogeneity among studies, *P* for heterogeneity was not significant for AA *vs* GG genotypes (Table 2).

### *TNF-A* −238

**Figure 2:**
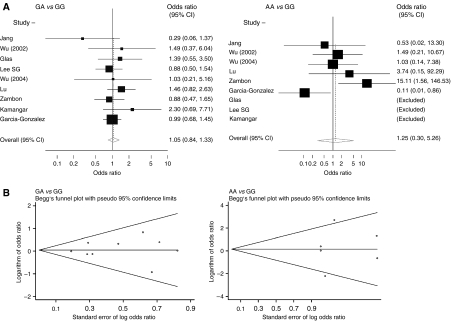
The association between *TNF-A* −238 and gastric cancer (GA and AA genotypes *vs* GG). (**A**) Forest plot – studies are sorted in order of publication year. (**B**) Begg's funnel plots for the associations.

Figure 2A summarises the ORs and 95% CIs for the associations between *TNF-A* −238A carrying genotypes and gastric cancer risk. For all gastric cancers, the random-effect overall ORs (95% CIs) were 1.05 (0.84–1.33) for GA *vs* GG genotypes and 1.25 (0.30–5.26) for AA *vs* GG genotypes. The fixed-effect ORs (95% CIs) were 1.04 (0.83–1.31) and 0.87 (0.41–1.87), respectively. When we investigated the inheritance models, *TNF-A* −238 showed neither recessive nor dominant models (data not shown). Therefore, the results are reported separately as GA *vs* GG and AA *vs* GG genotypes.

For noncardia cancers, the ORs (95% CIs) were 1.04 (0.71–1.52) for GA genotype and 1.38 (0.01–157.0) for AA genotype. Limiting the results to intestinal-type cancers, the ORs (95% CIs) were 1.40 (0.93–2.10) for GA genotype and 0.13 (0.01–2.20) for AA genotype. For this last comparison, one of the three studies was excluded because of the null values for AA genotype frequency among both cases and controls. For studies from western countries, summary ORs (95% CIs) for GA and AA genotypes were 1.05 (0.78–1.41) and 1.25 (0.01–171.9), respectively. For the studies from East Asian countries, these corresponding ORs (95% CIs) were 1.04 (0.69–1.58) and 1.28 (0.39–4.21), respectively.

In three studies, *TNF-A* −238 genotype in control subjects were not in HWE (Table 1). After excluding these three studies, the summary ORs (95% CIs) for GA and AA genotypes were 1.08 (0.75–1.55) and 4.04 (0.57–28.8), respectively.

Begg's funnel plot for the association between *TNF-A* −238 and gastric cancer is shown in Figure 2B. For both GA *vs* GG and AA *vs* GG genotypes, there was no evidence for bias using either the method of Begg and Mazumdar (*P* for rank correlation=1.00 and 0.85, respectively) or Egger's weighted regression method (*P* for bias=0.84 and 0.75, respectively). For GA genotype, after excluding studies with an SE>0.5, the summary OR (95% CI) was 1.04 (0.82–1.33). For AA genotype, all of the studies had an SE>0.5.

For GA genotype, the *Q*-statistic was nonsignificant for the overall association (*P*=0.55) and subgroup analyses, and *I*^*2*^-statistic showed little heterogeneity among studies (Table 2). For AA genotype, the *Q*-statistic was significant for the overall association (*P*=0.05) and also for noncardia, intestinal-type, and western populations subgroups, where *I*^*2*^-statistic showed moderate-to-high heterogeneity.

### *TNF-A* −857

**Figure 3:**
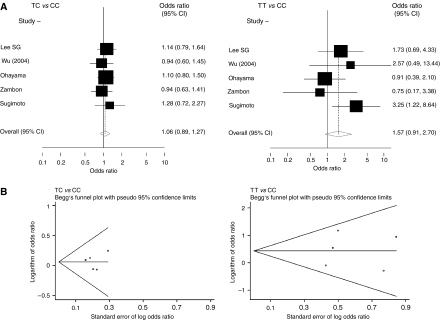
The association between *TNF-A* −857 and gastric cancer (TC and TT genotypes *vs* CC). (**A**) Forest plot – studies are sorted in order of publication year. (**B**) Begg's funnel plots for the associations.

Figure 3A summarises the ORs and 95% CIs for the associations between *TNF-A* −857T carrying genotypes (TC and TT *vs* CC) and gastric cancer risk. For all gastric cancers, the random-effect overall ORs (95% CIs) for TC *vs* CC and TT *vs* CC genotypes were 1.06 (0.89–1.27) and 1.57 (0.91–2.70), respectively. The fixed-effect ORs (95% CIs) were 1.06 (0.89–1.27) and 1.54 (0.98–2.43), respectively. Like *TNF-A* −238, *TNF-A* −857 showed neither recessive nor dominant model. Therefore, the results are reported separately for TC *vs* CC and TT *vs* CC genotypes.

Only one study reported the associations between *TNF-A* −857 and noncardia cancers. This study was also the only study from western countries; the ORs (95% CIs) for TC and TT genotypes in this study were 0.94 (0.63–1.41) and 0.75 (0.17–3.38), respectively. For studies from eastern countries, summary ORs (95% CIs) for TC and TT genotypes were 1.09 (0.90–1.33) and 1.73 (0.95–3.14), respectively.

In one study, *TNF-A* −857 genotypes among controls were not in HWE (Table 1). After excluding that study, the summary ORs (95% CIs) for TC and TT genotypes were 1.09 (0.90–1.33) and 1.73 (0.95–3.14), respectively.

Begg's funnel plot for the association between *TNF-A* −857 and gastric cancer is shown in Figure 3B. For both TC and CC genotypes, there was no evidence for bias using either the method of Begg and Mazumdar (*P* for rank correlation=0.81 and 0.46, respectively) or Egger's weighted regression method (*P* for bias=0.87 and 0.89, respectively). For TC *vs* CC genotypes, none of the studies had an SE>0.5. For TT *vs* CC genotypes, after excluding two studies with SEs>0.5, the summary OR (95% CI) changed to 1.66 (0.81–3.42).

The *Q*-statistic was nonsignificant for the overall associations and also for the subgroup analyses. Furthermore, *I*^*2*^-statistic showed little to moderate heterogeneity among studies (Table 2).

## DISCUSSION

Gastric cancer is the second most common cause of cancer death in the world (Parkin *et al*, 2005; Kamangar *et al*, 2006c). Because inflammation is one of the initial phases of gastric carcinogenesis, especially for intestinal-type gastric cancer (Correa, 1992), inflammation-related polymorphisms, including single-nucleotide polymorphisms (SNPs) in *TNF-A* gene, have been extensively studied in relation to gastric cancer (Camargo *et al*, 2006). The most extensively studied of these proinflammatory polymorphisms are two linked SNPs in *IL-1B* (−511C>T and −31T>C) and a penta-allelic variable number tandem-repeat polymorphism and allele 2 (IL-1RN^*^2). So far, at least three meta-analyses of the associations between these polymorphisms and gastric cancer risk have been published (Camargo *et al*, 2006; Kamangar *et al*, 2006b; Wang *et al*, 2007). However, to our knowledge, no systematic review has been previously published on the association between *TNF-A* SNPs and gastric cancer. Of the three polymorphisms reviewed in this report, –308G>A is studied more extensively and a biologic role for it has been identified.

An analysis of the 23 studies that presented results on *TNF-A* −308G>A showed no association between GA genotype (*vs* GG) and gastric cancer risk. However, there was a statistically significant increased risk associated with AA genotype using both fixed-effects and random-effects models. This association was limited to studies from western countries, and no association was found in studies from East Asian countries. Previous studies have suggested that frequencies of genetic markers often shows high variations among various ethnic and racial groups (Garte *et al*, 2001; Ioannidis *et al*, 2004), whereas differences in genetic effects (in terms of ORs) are much less common (Ioannidis *et al*, 2004). This meta-analysis found that the median prevalence of *TNF-A* −308A carrier genotypes was almost twice as high in western as in East Asian populations (23.5 *vs* 13.4%). However, unlike what has been shown for most previous associations (Ioannidis *et al*, 2004), the OR associated with AA genotype showed a difference between western and East Asian studies. We cannot explain the reasons for this latter observed difference.

No SNPs have been consistently associated with gastric cancer risk (Gonzalez *et al*, 2002). Indeed, because several initially promising gene–disease associations gravitated towards null over time (Ioannidis *et al*, 2001; Kamangar *et al*, 2006b), it has been suggested that journals should take a cautious approach in publishing such associations (Ioannidis, 2006). However, as shown in the results and discussed below, within the studies from western countries, the association between *TNF-A* −308AA genotype and gastric cancer risk seems robust to many tests, including testing for publication bias, heterogeneity, and HWE. Interestingly, a study within the InterLymph Consortium found that this same polymorphism was consistently associated with an increased risk of diffuse large B-cell lymphoma with a comparable magnitude of association (Rothman *et al*, 2006); the ORs associated with GA and AA genotypes (*vs* GG genotype) were 1.29 and 1.65, respectively. The studies that participated in this Consortium were all from western countries.

The forest plots for *TNF-A* −308AA genotype did not suggest a dominant effect for any single study. We used funnel plots and two formal statistical methods (Egger's weighted regression method and the rank correlation method of Begg and Mazumdar) to detect bias. In general, smaller studies, that is, those with higher SEs, had lower ORs, and there was some evidence for publication bias using both formal methods. However, excluding smaller studies did not materially change the results. In 20 out of 23 studies, distribution of *TNF-A* −308 among controls was in HWE. Limiting the analyses to these 20 studies, the results remained essentially unchanged. The overall heterogeneity between the studies was very low, as indicated by the *I*^*2*^ value of zero.

Since subgroup analyses are often limited by selective reporting of significant subgroup results, such analyses generally need to be interpreted with caution. However, in this report, limiting the results of *TNF-A* −308 polymorphism to noncardia gastric cancers or intestinal-type cancers made little difference. The summary ORs for the AG genotype remained close to null and were nonsignificant, whereas those for the GG genotype remained statistically significantly above one.

We found nine studies that examined the association between *TNF-A* −238G>A polymorphism and gastric cancer risk. Unlike that for *TNF-A* −308G>A polymorphism, a clear biologic role for this polymorphism has not been found (Jang *et al*, 2001). There was no association between gastric cancer risk and either the GA or the AA genotypes using either random-effects or fixed-effects models. Examining the data by anatomic and histologic subtypes did not make a difference. No statistically significant association was found when data were limited to East Asian populations, western populations, studies with larger sample sizes, or studies in which genotype frequencies were in HWE.

We found only five studies that had examined the association between *TNF-A* −857C>T polymorphisms and gastric cancer. No evidence was found for the association between CT (*vs* CC) genotype and gastric cancer. Almost all studies showed null associations, and there was little evidence for heterogeneity. The overall association with the TT genotype, however, was near significant. The overall pattern and magnitude of association is similar to that found for *TNF-A* −308G>A polymorphism, but further studies are needed to examine whether gastric cancer is significantly associated with this polymorphism. The studies showed some heterogeneity, with three showing strong positive associations, one showing no association, and one showing an inverse association. We believe that the currently available data do not provide conclusive evidence for the presence of an association, or lack thereof, between *TNF-A* −857TT genotype and gastric cancer.

Inappropriate selection of controls is a major source of bias in case–control studies. However, control groups for most of the studies used in this meta-analysis were selected from among healthy volunteers or blood donors, and *TNF-A* polymorphisms are unlikely to be associated with these conditions. Because *TNF* locus is on chromosome 6 (and not sex chromosomes), the distribution of this polymorphism is not associated with sex. Therefore, in theory, matching for sex should not affect the results.

Strengths of this meta-analysis are including 24 published studies with a large number of cases and controls, presenting data on several relevant methodologic aspects of these studies, subgroup analyses according to predefined criteria, and using other methods to examine the robustness of the summary statistics. We acknowledge that this meta-analysis also has limitations. Combining observational studies conducted in different populations with various qualities of design to obtain summary ORs and 95% CIs can sometimes be misleading (Shapiro, 1994), and summary statistics need to be interpreted with caution (Egger *et al*, 1998). However, as mentioned above, there is little evidence for improper selection of control groups or for associations within specific subgroups.

In summary, this systematic review found that *TNF-A* −308AA genotype was moderately associated with an increased risk of gastric cancer. *TNF-A* –308AG, *TNF-A* −238AA or AG, and *TNF-A* −857CT or TT were not statistically significantly associated with gastric cancer risk. It is possible that, with increasing the number of studies, *TNF-A* −857TT may also be associated with an increased risk of gastric cancer.

## Figures and Tables

**Table 1:** Study characteristics

	**First author**	**Study location**	**Year^a^**	**Number of cases/controls**	**Source of control selection**	**% −308A allele frequency^b^**	**% −238A allele frequency^c^**	**% −857T allele frequency^d^**	***P*, HWE −308^e^**	***P*, HWE −238^f^**	***P*, HWE −857^g^**
1	Jang	Korea (E)	2001	52/92	Healthy volunteers	3.8	7.1	—	0.70	0.39	—
2	Wu	Taiwan (E)	2002	150/220	Healthy volunteers	12.0	1.8	—	<0.01	<0.01	—
3	El-Omar	USA (W)	2003	314/210	Population based	15.2	—	—	0.55	—	—
4	Machado	Portugal (W)	2003	287/304	Healthy volunteers	12.7	—	—	0.65	—	—
5	Fei	China (E)	2004	56/164	Healthy volunteers	6.7	—	—	0.12	—	—
6	Glas	Germany (W)	2004	88/145	Healthy volunteers	15.2	3.8	—	0.67	0.63	—
7	Lee SG	Korea (E)	2004	341/261	Healthy volunteers	8.4	4.8	15.9	0.49	0.42	0.85
8	Torres	Colombia (W)	2004	44/66	Clinic based	7.6	—	—	0.51	—	—
9	Wu	Taiwan (E)	2004	204/210	Healthy volunteers	12.4	1.7	14.3	<0.01	<0.01	0.20
10	Ohayama	Japan (E)	2004	300/472	Clinic based	—	—	18.6	—	—	0.90
11	Garza-Gonzalez	Mexico (W)	2005	63/215	Clinic based	8.6	—	—	0.61	—	—
12	Guo	China (E)	2005	264/437	Healthy volunteers	5.9	—	—	<0.01	—	—
13	Lee JY	Korea (E)	2005	122/120	Healthy volunteers	7.1	—	—	0.40	—	—
14	Li	China (E)	2005	59/264	Healthy volunteers	7.2	—	—	0.56	—	—
15	Lu	China (E)	2005	250/300	Population based	4.7	3.8	—	0.08	0.49	—
16	Perri	Italy (W)	2005	184/362	Healthy volunteers	10.9	—	—	0.15	—	—
17	Rocha	Brazil (W)	2005	161/535	Healthy volunteers	13.9	—	—	0.34	—	—
18	Zambon	Italy (W)	2005	129/644	Clinic based	12.3	5.9	19.6	0.90	0.38	<0.01
19	Kamangar	Finland (W)	2006	112/208	Healthy cohort subjects	13.5	1.2	—	0.29	0.86	—
20	Kim	Korea (E)	2006	237/461	Healthy volunteers	6.8	—	—	0.91	—	—
21	Morgan	Honduras (W)	2006	168/161	Population based	3.7	—	—	0.62	—	—
22	Hou	Poland (W)	2007	305/427	Population based	16.2	—	—	0.19	—	—
23	Sugimoto	Japan (E)	2007	105/172	Clinic based	0.9	—	15.7	0.91	—	0.11
24	Garcia-Gonzalez	Spain (W)	2007	404/404	Healthy volunteers	11.3	10.3	—	0.35	0.01	—

E=East Asian country; HWE=Hardy–Weinberg equilibrium; TNF-A=tumour-necrosis factor-A; W=western country.

a Year: Publication year.

b Percentage of *TNF-A* –308A allele frequency among controls.

c Percentage of *TNF-A* –238 A allele frequency among controls.

d Percentage of *TNF-A* –857 T allele frequency among controls.

e *P*-value for HWE for *TNF-A* –308 polymorphism among controls.

f *P*-value for HWE for *TNF-A* –238 polymorphism among controls.

g *P*-value for HWE for *TNF-A* –857 polymorphism among controls.

**Table 2:** Overall and group-specific summary statistics for *TNF-A* −308, *TNF-A* −238, and *TNF-A* −857 and gastric cancer

	**Number of studies**	**Polymorphisms**	***Q*-statistic^a^**	***P*-value^b^**	***I*^2^ (%)^c^**	**Random-effect OR (95% CI)**	**Fixed-effect OR (95% CI)**
*TNF-A –308 (4099/6383)* d							
All gastric cancers	23	GA *vs* GG	36.43	0.03	40	1.09 (0.94–1.27)	1.14 (1.02–1.27)
	19^e^	AA *vs* GG	13.77	0.74	0	1.49 (1.11–1.99)	1.51 (1.14–1.99)
Noncardia cancers	6	GA *vs* GG	11.80	0.04	58	1.17 (0.85–1.59)	1.23 (1.02–1.49)
	6	AA *vs* GG	4.18	0.52	0	2.88 (1.74–4.77)	2.97 (1.83–4.84)
Intestinal-type cancers	6	GA *vs* GG	11.68	0.04	57	0.98 (0.68–1.40)	1.03 (0.83–1.29)
	6	AA *vs* GG	4.08	0.44	0	2.17 (1.17–4.03)	2.00 (1.11–3.61)
*Helicobacter pylori*-positive subjects	3	GA *vs* GG	4.03	0.13	50	0.66 (0.33–1.30)	0.77 (0.51–1.15)
	2^e^	AA *vs* GG	2.63	0.11	62	3.23 (0.10–99.84)	4.05 (0.91–18.04)
Western populations	12	GA *vs* GG	18.37	0.07	40	1.14 (0.95–1.37)	1.19 (1.04–1.36)
	10^e^	AA *vs* GG	6.53	0.69	0	1.74 (1.21–2.51)	1.76 (1.24–2.50)
East Asian population	11	GA *vs* GG	16.80	0.08	40	1.02 (0.78–1.34)	1.03 (0.85–1.25)
	9^e^	AA *vs* GG	5.36	0.72	0	1.14 (0.70–1.84)	1.15 (0.72–1.83)
Studies in HWE	20	GA *vs* GG	33.83	0.02	44	1.08 (0.91–1.28)	1.14 (1.01–1.28)
	16^e^	AA *vs* GG	11.28	0.73	0	1.73 (1.22–2.44)	1.73 (1.25–2.41)
							
*TNF-A –238 (1730/2484)* d							
All gastric cancers	9	GA *vs* GG	6.87	0.55	0	1.05 (0.84–1.33)	1.04 (0.83–1.31)
	6^e^	AA *vs* GG	11.12	0.05	55	1.25 (0.30–5.26)	0.87 (0.41–1.87)
Noncardia cancers	3	GA *vs* GG	2.39	0.30	16	1.04 (0.71–1.52)	1.01 (0.74–1.40)
	2^e^	AA *vs* GG	9.49	<0.01	89	1.38 (0.01–157.0)	0.72 (0.24–2.09)
Intestinal-type cancers	3	GA *vs* GG	1.80	0.41	0	1.40 (0.93–2.10)	1.39 (0.93–2.08)
	2^e^	AA *vs* GG	0.00	—	0	0.13 (0.01–2.20)	0.13 (0.01–2.20)
*H. pylori*-positive subjects	0	—	—	—	—	—	—
Western populations	4	GA *vs* GG	2.33	0.51	0	1.05 (0.78–1.41)	1.05 (0.78–1.41)
	2^e^	AA *vs* GG	10.25	<0.01	90	1.25 (0.01–171.9)	0.64 (0.22–1.85)
East Asian populations	5	GA *vs* GG	4.54	0.34	12	1.04 (0.69–1.58)	1.03 (0.72–1.47)
	4^e^	AA *vs* GG	0.79	0.85	0	1.28 (0.39–4.21)	1.30 (0.41–4.06)
Studies in HWE	6	GA *vs* GG	6.53	0.26	23	1.08 (0.75–1.55)	1.06 (0.78–1.42)
	3^e^	AA *vs* GG	2.85	0.24	30	4.04 (0.57–28.8)	3.79 (0.93–15.40)
							
*TNF-A –857 (1079/1759)* d							
All gastric cancers	5	CT *vs* CC	1.24	0.87	0	1.06 (0.89–1.27)	1.06 (0.89–1.27)
	5	TT *vs* CC	5.06	0.28	21	1.57 (0.91–2.70)	1.54 (0.98–2.43)
Noncardia cancers	1	CT *vs* CC	0.00	—	0	0.94 (0.63–1.41)	0.94 (0.63–1.41)
	1	TT *vs* CC	0.00	—	0	0.75 (0.17–3.38)	0.74 (0.17–3.38)
Intestinal-type cancers	0	—	—	—	—	—	—
*H. pylori*-positive subjects	0	—	—	—	—	—	—
Western populations	1	CT *vs* CC	0.00	—	0	0.94 (0.63–1.41)	0.94 (0.63–1.41)
	1	TT *vs* CC	0.00	—	0	0.75 (0.17–3.38)	0.75 (0.17–3.38)
East Asian populations	4	CT *vs* CC	0.81	0.85	0	1.09 (0.90–1.33)	1.09 (0.90–1.33)
	4	TT *vs* CC	4.07	0.25	26	1.73 (0.95–3.14)	1.68 (1.04–2.72)
Studies in HWE	4	CT *vs* CC	0.81	0.85	0	1.09 (0.90–1.33)	1.09 (0.90–1.33)
	4	TT *vs* CC	4.07	0.25	26	1.73 (0.95–3.14)	1.68 (1.04–2.72)

CI=confidence interval; HWE=Hardy–Weinberg equilibrium; OR=odds ratio; TNF-A=tumour-necrosis factor-A.

a *χ*^2^ *Q*-statistic for homogeneity in random-effect model.

b *P*-value for the *Q*-statistic in random-effect model.

c Higgins' *I^2^*-statistic for heterogeneity in random-effect model.

d Number of cases/number of controls.

e Some studies were excluded because of the null values for AA genotype frequency among both cases and controls.
